# Lack of Effects of Renin-Angiotensin-Aldosterone System Activity and Beta-Adrenoceptor Pathway Polymorphisms on the Response to Bisoprolol in Hypertension

**DOI:** 10.3389/fcvm.2022.842875

**Published:** 2022-04-01

**Authors:** Weiwei Zeng, Tanya T. W. Chu, Chung Shun Ho, Clara W. S. Lo, Alan S. L. Chan, Alice P. S. Kong, Brian Tomlinson, Sze Wa Chan

**Affiliations:** ^1^Shenzhen Baoan Women’s and Children’s Hospital, Jinan University, Shenzhen, China; ^2^Department of Medicine and Therapeutics, Prince of Wales Hospital, The Chinese University of Hong Kong, Shatin, Hong Kong SAR, China; ^3^Department of Chemical Pathology, Prince of Wales Hospital, The Chinese University of Hong Kong, Shatin, Hong Kong SAR, China; ^4^Faculty of Medicine, Macau University of Science and Technology, Macau, Macau SAR, China; ^5^School of Health Sciences, Caritas Institute of Higher Education, Hong Kong, Hong Kong SAR, China

**Keywords:** β1-adrenoceptor polymorphism, bisoprolol, blood pressure, renin, aldosterone, angiotensin II

## Abstract

**Purpose:**

This study examined the effects of plasma renin activity (PRA), angiotensin II (Ang II) and aldosterone (PAC) concentrations as well as common polymorphisms in the β_1_-Adrenoceptor gene (*ADRB1*) and the G-protein α-Subunit (G_αs_) protein gene the G protein α-Subunit 1 gene (*GNAS*) on the blood pressure (BP) and heart rate (HR) response to bisoprolol in Chinese patients with hypertension.

**Methods:**

Patients with sitting clinic systolic BP (SBP) 140–169 mmHg and/or diastolic BP (DBP) 90–109 mmHg after placebo run-in were treated with open-label bisoprolol 2.5 mg daily for 6 weeks. Patients diagnosed as having primary aldosteronism or renal artery stenosis were excluded. PRA, Ang II and PAC concentrations were measured after the placebo run-in and after 6 weeks of treatment. The Ser49Gly and Arg389Gly polymorphisms in *ADRB1* and the c.393C > T polymorphism in *GNAS* were genotyped by the TaqMan^®^ assay.

**Results:**

In 99 patients who completed the study, baseline PAC levels were significantly associated with baseline DBP and plasma potassium on univariate but not on multivariate linear regression analysis. PRA, Ang II, and PAC concentrations at baseline were not associated with changes in BP with bisoprolol treatment, but the values were all significantly reduced (PRA −0.141 ± 0.595 ng/mL/h, Ang II −2.390 ± 5.171 pmol/L and aldosterone −51.86 ± 119.1 pg/mL; all *P* < 0.05) following 6 weeks of bisoprolol treatment. There were no significant differences in BP or HR responses in patients with baseline PRA above or below the PRA cut-point of 0.65 ng/mL/h or the median value of 0.9 ng/ml/hour. There were no significant associations of the *ADRB1* and *GNAS* polymorphisms with the clinic and ambulatory BP and HR responses to bisoprolol.

**Conclusion:**

Baseline PRA, PAC and Ang II concentrations showed no significant association with the BP response to bisoprolol treatment, but all these parameters were reduced after 6 weeks of treatment with bisoprolol. The two common polymorphisms in *ADRB1* and the c.393C > T polymorphism in *GNAS* had no significant association with the BP and HR response to bisoprolol in these patients.

## Introduction

High blood pressure (BP) is highly prevalent worldwide, and it is the major risk factor for ischemic and haemorrhagic stroke and an important risk factor for coronary heart disease, heart failure and chronic kidney disease (1–3). According to international guidelines, BP control is only achieved in a relatively small proportion of patients (4, 5). For any particular class of antihypertensive drugs, BP responses vary among individuals due to differences in clinical characteristics (e.g., age, body mass index) (6, 7) and genetic factors (8).

The renin-angiotensin-aldosterone system (RAAS) is closely related to vasomotor control and water and salt homeostasis in the body. It plays a crucial role in developing hypertension (9). Plasma renin, angiotensin II and aldosterone directly affect the proliferation of arterial endothelial cells and participate in the pathological process of hypertensive arterial damage (10).

Beta-adrenoceptor antagonists (β-Blockers) represent an important class of cardiovascular medications for managing hypertension and reducing morbidity and mortality in patients with heart failure, angina, and after myocardial infarction (11, 12). Bisoprolol is a moderately lipophilic and highly β1-Selective adrenoceptor antagonist devoid of intrinsic sympathomimetic activity (ISA), vasodilatory effects or membrane-stabilizing properties. The antihypertensive effects of some antihypertensive drugs, including β-Blockers, may be related to an effect on the RAAS and a reduction in the sympathetic tone and baseline activity of these systems may influence the response (13, 14). It has been suggested that measuring plasma renin activity (PRA) may be helpful to predict the BP response to certain drugs (15), but other reports considered it not useful (16). A recent study suggested PRA may be useful to predict BP responses in European Americans but not in African Americans, and European Americans with PRA ≥ 0.65 ng/ml/hour showed a better response to a β-Blocker than to a diuretic (17).

β-Adrenergic receptor polymorphisms may influence responses to β-Blocker treatments (18). It has been demonstrated that the Arg16Gly and Gln27Glu β2-Adrenoceptor polymorphisms were associated with changes in triglyceride levels over time following carvedilol or metoprolol succinate treatments (19). The β1-Adrenoceptor exists in two agonist conformations – a high-affinity catecholamine conformation and a low-affinity secondary agonist conformation. The polymorphic variants may affect receptor-effector coupling and intracellular signaling from the different conformations. The two common polymorphisms, 145A > G (rs1801252, Ser49Gly) and 1165G > C (rs1801253, Arg389Gly) within the human β1-Adrenoceptor gene (ADRB1), have been reported to be associated with reduced BP or heart rate (HR) responses to β-Blockers (20–22).

The stimulatory G protein subtype Gs is a trimeric transmembrane protein that mediates the signals from β1-Adrenoceptors to adenylyl cyclase, which catalyzes the production of the second-messenger cAMP. The G protein α-Subunit 1 gene (GNAS) in chromosome 20 (20q13.2) encodes the α-Subunit that couples β1-Adrenoceptors with adenylyl cyclase. The synonymous c.393C > T (rs7121) polymorphism in this gene has been reported to be associated with autonomic nervous system (ANS) dysfunction and to modulate BP and HR responses to exercise or β-Blocker treatment (23–25). It has also been shown to be a marker for disease progression and survival in certain cancers (26).

The present study aimed to investigate the effects of baseline PRA, angiotensin II (Ang II) and plasma aldosterone (PAC) concentrations on the response to bisoprolol and the effect of bisoprolol treatment on these parameters in Chinese patients with primary hypertension. We also examined the effects of common polymorphisms in ADRB1 and GNAS on the clinic and ambulatory BP and HR responses to bisoprolol.

## Materials and Methods

### Patients

Patients with primary hypertension identified from outpatient clinics at the Prince of Wales Hospital, Hong Kong, were enrolled in the study. The study involving human participants was reviewed and approved by the Joint Clinical Research Ethics Committee of the Chinese University of Hong Kong and New Territories East Cluster (CUHK-NTEC) with reference number CRE-2011.616-T. The study was performed following the ethical standards in the Declaration of Helsinki and subsequent revisions. All patients signed the Informed Consent.

The inclusion and exclusion criteria have been described previously (27). Briefly, subjects with sitting clinic systolic BP (SBP) 140–169 mmHg and/or diastolic BP (DBP) 90–109 mmHg in patients without type 2 diabetes (T2D) or SBP 130–169 mmHg and/or DBP of 80–109 mmHg in those patients with T2D after placebo run-in were treated with open-label bisoprolol 2.5 mg daily for 6 weeks. Other antihypertensive treatments were discontinued except for amlodipine which was continued if necessary to achieve BPs in the defined range at the end of the run-in. Patients diagnosed as having primary aldosteronism or renal artery stenosis were excluded.

### Study Design

The study participants consisted of two groups with 50 subjects in each group, according to the total duration of bisoprolol treatment. The study protocol has been described previously (27). In group A, subjects were given bisoprolol 2.5 mg once daily for 6 weeks. Venous blood samples were collected after the placebo run-in to determine PRA, Ang II and PAC. In group B, subjects were treated with bisoprolol 2.5 mg once daily for 6 weeks and then continued treatment for a total of 24 weeks with optional titration of the dose of bisoprolol by doubling the dose after 6-week intervals up to 10 mg to achieve target BP levels. Venous blood samples were collected after the placebo run-in and after 6 weeks of treatment. For both groups, clinic BP and 24-hr ABP measurements were made at baseline and at the end of 6 weeks’ treatment with bisoprolol 2.5 mg. ABP measurements were taken using a wrist-type (BPro, HealthSTATS International, Singapore) or arm-type (A&D TM-2430, Tokyo, Japan) ABP device for 24 h, and the BPs were measured at intervals automatically throughout 24 h (27). All patients in group A completed the study while one patient from group B did not complete the 24-week treatment.

### Measurement of Renin-Angiotensin-Aldosterone System Biomarkers by Liquid Chromatography-Tandem Mass Spectrometry Methods

#### Materials

The aldosterone standard was from Toronto Research Chemical Inc. (Toronto, ON, Canada), and the aldosterone_d7 internal stand (IS) was from IsoSciences (Ambler, PA, United States). Angiotensin I (Ang I), Ang I internal standard (Ang I IS) [DR[V^13^C,^15^N]YIHPFHL], and Ang I degradation standard (DS) [DR[V^13^C,^15^N]Y[I^13^C,^15^N]HPFHL] were from Anaspec (Fremont, CA, United States). Aminoethyl benzene sulfonyl fluoride (AEBSF) hydrochloride was from ACROS Organics (Thermo Fisher Scientific, Branchburg, NJ, United States). Ammonium acetate, maleic acid, and tert-butyl methyl ether (MBTE) were from Sigma Aldrich (St Louis, MI, United States). LCMS grade acetonitrile, methanol, and water were from Thermo Fisher Scientific. Formic acid was from BDH (Radnor, PA, United States). Phosphate buffered saline (PBS) was purchased from Sigma-Aldrich (St. Louis, MO, United States).

#### Measurement of PAC

The quantitation of PAC was by an electrospray negative ionization LC-MS/MS after organic solvent extraction of PAC from EDTA plasma samples. Two hundred microliters of EDTA plasma and IS was added to 1.2 mL MBTE and vortex-mixed for 10 min to extract PAC. The mixture was centrifuged and frozen at −80°C for at least 1 h. The supernatant was dried at 45°C, and the dried residue was reconstituted with 120 μL of 90% methanol. The solution was washed with 1 mL hexane to reduce matrix interference. The upper hexane layer was discarded, and the lower layer containing PAC and PAC IS dried at 45°C in a centrifugal evaporator (Savant SpeedVac Concentrator from Thermo Fisher Scientific). The dried extract was reconstituted with 120 μL 10% acetonitrile and was ready for LC-MS/MS analysis.

Separation of matrix interferences from PAC and its IS was on a Waters BEH C18 column (100 × 2.1 mm, 1.7 μm) (Waters, Milford, MA, United States) by gradient programming of two mobile phase solutions (A: 1 mM ammonium acetate in water and B: acetonitrile). PAC calibrators were prepared in 1% acetonitrile solution. The Waters ACQUITY Xevo-TQ system was used to detect PAC and IS. The quantitative analysis of PAC and IS was performed by monitoring the MRM transitions: *m/z* 359→331 and *m/z* 366→338, respectively. An additional MRM of PAC at *m/z* 359→189 was used as a qualitative MRM. Results calculated from qualifying MRM should differ from quantifying MRM by less than 20%. Waters MassLynx version 4.1 was used for data acquisition and processing. The relative peak area response ratios of PAC to IS were plotted against the corresponding calibrator concentrations with 1/× weighting to set up a calibration curve on the Waters TargetLynx version 4.1.

The analytical measuring range of this method was 50–5200 pmol/L with a between-batch coefficient of variation (CV) <5%. There was no significant carryover or matrix interference.

#### Measurement of PRA

PRA measurement in EDTA plasma samples was by electrospray positive LC-MS/MS. The increase in Ang I calculated the PRA after incubation of two aliquots from the same plasma sample at 4 and 37°C for 3 h in the presence of AEBSF, a serine protease inhibitor. The generation of Ang I was automated on a liquid handler workstation (Perkin Elmer Janus, Waltham, MA, United States). DS was included in the generation cocktail to monitor the presence of endogenous peptidases that could degrade the generated Ang I. The addition of acetonitrile terminated the PRA enzymatic reaction. Plasma protein was removed after centrifugation. The supernatant was acidified with 10% formic acid, and Ang I-IS was added to each aliquot on a 96-well plate. The LC-MS/MS measurement was performed on a Waters ACQUITY Xevo-TQ system. Ang I and Ang I IS were first purified by online solid-phase extraction using Waters Oasis HLB cartridge column (3.9 × 20 mm, 15 μm). Further separation of matrix interference was on a Waters Xbridge C18 column (2.1 × 50 mm, 5 μm) by gradient programming of two mobile phase solutions (A: 0.1% formic acid in LCMS water; and B: 0.1% formic acid in 20/80 acetonitrile/methanol). Ang I, IS, and DS were detected by monitoring the MRM transitions: *m*/*z* 433→534, 437→660, and 435→653, respectively. An additional MRM of Ang I at *m*/*z* 433→619 was used as a qualitative MRM. Ang I calibrators were prepared in 50% acetonitrile. Quantitation of Ang I on the Waters MassLynx and TargetLynx managers was similar to those described for PAC. The analytical measuring range of this method was 0.07–183 ng/mL/h with a between-batch CV <8%. There was no significant carryover or matrix interference.

#### Measurement of Ang II

The LC-MS/MS method to measure EDTA plasma Ang II was described by Lo et al. (28).

### Biochemical Assessments

Measurements of total cholesterol, high-density lipoprotein cholesterol (HDL-C), triglyceride (TG), fasting plasma glucose (FPG), and glycosylated hemoglobin (HbA1c) have been described previously (27). The low-density lipoprotein cholesterol (LDL-C) level was estimated using the Friedewald formula (29) or directly measured when the TG level was over 4.5 mmol/L.

### Genotyping

Two common polymorphisms in ADRB1 [49A > G (rs1801252), 389C > G (rs1801253)] and the 393C > T (rs7121) polymorphism in GNAS were selected in this study. DNA was extracted from peripheral whole blood samples by the phenol-chloroform method. Genetic polymorphisms in ADRB1 and GNAS were genotyped by the TaqMan^®^ assay using the geneAmp PCR system 9700 (Applied Biosystems, Foster City, CA, United States). The polymorphisms were determined using a previously reported polymerase chain reaction (PCR) restriction fragment length polymorphism (RFLP) method (26). All polymorphisms examined in this study were in Hardy–Weinberg equilibrium (χ^2^ test *P* > 0.05), and the frequencies of the minor alleles were similar to those reported in Han Chinese in HapMap.

### Power Calculation

Using the Arg389Gly (389Arg allele frequency 0.761 in Chinese Han) and Ser49Gly (49Gly frequency 0.161 in Chinese Han) polymorphisms in ADRB1 gene, a two group Chi-square test with a 0.05 two-sided significance level will have 80% power to detect a 10% difference of DBP between the reference group and the affected group after 6 weeks of treatment when sample size is 75. The present studies recruited 100 subjects which is sufficient to detect the relevant differences based on the allele frequencies.

### Statistical Analysis

All statistical analyses were performed using SPSS software (Version 26, SPSS Inc., IBM Company, Chicago, IL, United States). Data were pooled from two open-label, placebo run-in pharmacogenetic studies of bisoprolol treatment. The distribution of continuous data was evaluated according to the Shapiro–Wilk test. Logistic regression analyses were applied to determine significantly independent predictors of PRA, PAC and Ang II levels. Correlations between baseline PRA, PAC, and Ang II and changes in BP and HR were assessed using Pearson correlation coefficient analysis. BP and HR responses were compared in subjects with PRA values below and ≥0.65 ng/ml/h and between subjects above and below the median value. Skewed data were log transformed. Statistical analysis on the effect of bisoprolol treatment on PRA, Ang II and PAC was performed using a paired-sample *t*-test or Wilcoxon signed-rank test, as appropriate. Statistical analysis on the effect of genetic polymorphisms on the BP and HR responses to bisoprolol was performed using an independent samples *t*-test. Data are presented as mean ± standard deviation unless otherwise specified. A *P*-value <0.05 was considered statistically significant.

## Results

### Study Population

**FIGURE 1:**
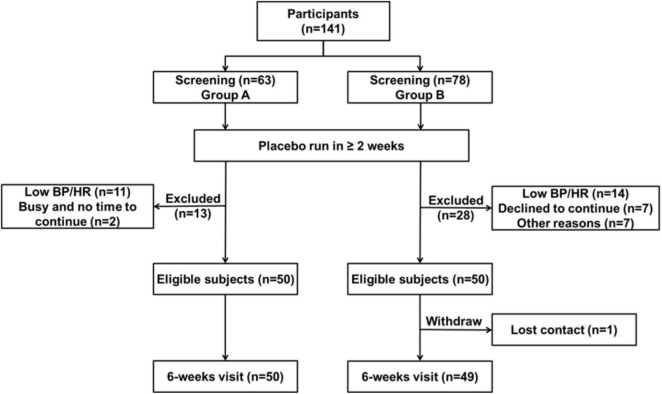
Consort diagram of the study.

From 141 patients who underwent screening, 99 subjects of Chinese ethnicity completed the studies (Figure 1). The data from the two studies were combined in further analysis (27). The demographic and baseline characteristics and concomitant diseases of the study patients are shown in Table 1. Subjects were generally overweight by Asian standards (median body weight of 66.2 kg, 25th percentile 57.5, 75th percentile 77.0 kg), with a median BMI of 25.1 kg/m2 (25th percentile 22.7, 75th percentile 27.9 kg/m2) with a mean age of 54 ± 10 years. The mean baseline PRA, Ang II, and PAC were 1.1 ng/mL/h, 14.0 pg/mL, and 213.8 pmol/L, respectively. The baseline clinic BPs were 144.1 ± 10.6 mmHg/92.2 ± 9.3 mmHg.

**TABLE 1:** Baseline characteristics of the study population.

Parameters	Baseline
N (% male)	99 (60%)
**N**	
Age (years)	54 ± 10
BMI (kg/m^2^)	25.1 (22.7–27.9)
Body weight (kg)	66.2 (57.5–77.0)
Current smoker	8 (8%)
Drinker	8 (8%)
Renin (ng/mL/h)	1.1 (0.6–1.7)
Angiotensin II (pg/mL)	14.0 (9.0–15.8)
Aldosterone (pmol/L)	213.9 (118.0–280.0)
Plasma potassium	3.9 ± 0.4
Plasma sodium	141.0 (140.0–142.0)
Clinic SBP (mmHg)	144.1 ± 10.6
Clinic DBP (mmHg)	92.2 ± 9.3
Clinic HR (beats/min)	71.6 ± 10.4
Ambulatory SBP (mmHg)	143.1 ± 11.3
Ambulatory DBP (mmHg)	92.1 ± 9.1
Ambulatory HR (beats/min)	74.8 ± 7.4
Daytime SBP (mmHg)	147.1 ± 11.5
Daytime DBP (mmHg)	94.8 ± 9.2
Daytime HR (beats/min)	77.9 ± 8.0
Nighttime SBP (mmHg)	132.7 ± 14.6
Nighttime DBP (mmHg)	84.3 ± 11.7
Nighttime HR (beats/min)	66.6 ± 8.0
**Medical history**	
Hyperlipidemia *(n)*	41
Diabetes *(n)*	13
**Medication record**	
With amlodipine *(n)*	21
Without amlodipine *(n)*	78

*Data are expressed as mean ± SD or median (25th percentile and 75th percentile). BMI, body mass index; SBP, systolic blood pressure; DBP, diastolic blood pressure; HR, heart rate.*

### Effects of Renin, Angiotensin II and Aldosterone on Blood Pressure and Heart Rate Responses to Bisoprolol and Effects of Bisoprolol Treatment on These Parameters

On univariate linear regression analysis, PAC levels were significantly associated with age (*P* = 0.026), baseline body weight (*P* = 0.040), baseline DBP (*P* = 0.024) and baseline plasma potassium (*P* = 0.027), but not sex, baseline SBP and sodium level. The multiple linear regression analysis failed to detect a significant association of any of these factors with PAC. Baseline Ang II levels were significantly associated with sex on univariate (*P* = 0.010) and multivariate (*P* = 0.008) linear regression analyses (Table 2). The PRA level was not significantly associated with any of these factors.

**TABLE 2:** Linear regression analysis for the factors that may influence plasma renin activity (PRA), aldosterone and angiotensin II concentrations.

	Univariate		Multivariate	
	B (95% CI for B)	*P*	B (95% CI for B)	*P*
**PRA (*n* = 73)**				
Sex	0.369(−0.089−0.882)	0.108	0.389(−0.239−1.017)	0.221
Age	−0.021(−0.044−0.002)	0.072	−0.020(−0.050−0.009)	0.179
Baseline bodyweight	0.010(−0.07−0.027)	0.237	−0.002(−0.025−0.020)	0.838
Baseline SBP	−0.001(−0.022−0.020)	0.924	0.004(−0.019−0.027)	0.739
Baseline DBP	0.017(−0.012−0.045)	0.247	0.005(−0.030−0.039)	0.788
Baseline plasma potassium	0.044(−0.594−0.683)	0.890	0.251(−0.434−0.936)	0.467
Baseline plasma sodium	−0.035(−0.172−0.102)	0.612	−0.016(−0.156−0.123)	0.817
**Aldosterone (*n* = 76)**				
Sex	41.671(−14.773−98.115)	0.145	2.172(−63.935−68.279)	0.948
Age	−3.054(−5.732−0.376	0.026	−0.062(−3.634−3.511)	0.973
Baseline bodyweight	2.133(0.103−4.164)	0.040	1.273(−1.288−3.833)	0.325
Baseline SBP	−1.347(−3.761−1.068)	0.270	−1.812(−4.277−0.653)	0.147
Baseline DBP	3.330(0.451−6.209)	0.024	2.443(−1.090−5.976)	0.172
Baseline plasma potassium	−84.903(−159.687−10.119)	0.027	−65.031(−145.689−15.627)	0.112
Baseline plasma sodium	−10.760(−24.575−3.055)	0.125	−10.298(−23.804−3.209)	0.133
**Angiotensin II (*n* = 64)**				
Sex	5.124(1.294−8.954)	0.010	6.404(1.707−11.101)	0.008
Age	−0.100(−0.284−0.085)	0.285	−0.164(−0.410−0.082)	0.186
Baseline bodyweight	0.055(−0.081−0.190)	0.424	−0.071(−0.240−0.098)	0.403
Baseline SBP	0.015(−0.155−0.184)	0.862	0.086(−0.089−0.261)	0.329
Baseline DBP	0.050(−0.156−0.256)	0.628	−0.060(−0.311−0.191)	0.634
Baseline plasma potassium	1.455(−3.808−6.718)	0.582	2.270(−3.424−7.964)	0.428
Baseline plasma sodium	−0.530(−1.524−0.464)	0.291	−0.287(−1.282−0.708)	0.565

After 6 weeks of bisoprolol treatment, change in clinic HR showed a weak but significant correlation with baseline PRA (*P* = 0.044), but not PAC and Ang II levels (Table 3). There was no significant correlation between ambulatory HR values changes or any changes in BP values with baseline PRA, PAC or Ang II levels. There was no significant difference in the BP or HR responses in subjects with PRA values below or ≥0.65 ng/ml/hour (number of patients with PRA <0.65 ng/ml/h = 27; ≥ 0.65 ng/ml/h = 46) or between subjects with PRA values above and below the median value of 0.90 (25th percentile 0.40, 75th percentile 1.69) ng/ml/hour (data not shown). These three RAAS biomarker levels were reduced significantly compared to baseline (PRA −0.141 ± 0.595 ng/mL/h, Ang II −2.390 ± 5.171 pmol/L and PAC −51.86 ± 119.1 pg/mL; all *P* < 0.05) following 6 weeks of bisoprolol treatment (Table 4).

**TABLE 3:** Correlations between baseline plasma renin activity (PRA), angiotensin II and aldosterone concentrations and changes in blood pressure and heart rate after 6 weeks of treatment with bisoprolol 2.5 mg.

Parameters	Baseline PRA		Baseline angiotensin II		Baseline aldosterone	
	Pearson’s R	*P*	Pearson’s R	*P*	Pearson’s R	*P*
Clinic SBP (mmHg)	−0.012	0.924	0.124	0.330	0.090	0.445
Clinic DBP (mmHg)	−0.162	0.175	0.066	0.604	−0.037	0.750
Clinic HR (beats/min)	−0.238	0.044	−0.025	0.842	0.005	0.965
Ambulatory SBP (mmHg)	−0.118	0.324	0.072	0.570	−0.139	0.233
Ambulatory DBP (mmHg)	−0.148	0.215	0.030	0.815	−0.177	0.129
Ambulatory HR (beats/min)	−0.055	0.643	0.172	0.175	−0.148	0.204
Daytime SBP (mmHg)	−0.115	0.335	0.052	0.683	−0.134	0.253
Daytime DBP (mmHg)	−0.155	0.193	−0.003	0.979	−0.176	0.131
Daytime HR (beats/min)	−0.063	0.599	0.129	0.308	−0.123	0.294
Night-time SBP (mmHg)	−0.171	0.152	0.048	0.707	−0.128	0.172
Night-time DBP (mmHg)	−0.218	0.066	0.046	0.721	−0.223	0.055
Night-time HR (beats/min)	−0.114	0.342	0.118	0.353	−0.128	0.275

*SBP, systolic blood pressure; DBP, diastolic blood pressure; HR, heart rate.*

**TABLE 4:** Effect of bisoprolol on plasma renin activity (PRA), angiotensin II and aldosterone after 6 weeks’ treatment.

	Baseline	6 weeks	Absolute change	*P-*value	P* value
PRA (ng/mL/h) (*n* = 37)	0.898 ± 0.709	0.757 ± 0.660	−0.141 ± 0.595	0.040	/
Angiotensin II (pg/mL) (*n* = 26)	12.35 ± 5.77	9.965 ± 3.912	−2.390 ± 5.171	0.023	0.056
Aldosterone (pmol/L) (*n* = 36)	229.1 ± 111.9	177.3 ± 78.09	−51.86 ± 119.1	0.017	/

Data were expressed as mean ± SD and analyzed by paired t-test or Wilcoxon Signed-Rank Test. P* value refers to comparison of the plasma angiotensin II confounded by sex.

### Effect of β_1_-Adrenoceptor Gene and the G Protein α-Subunit 1 Gene Polymorphisms on the Blood Pressure and Heart Rate Responses to Bisoprolol

**FIGURE 2:**
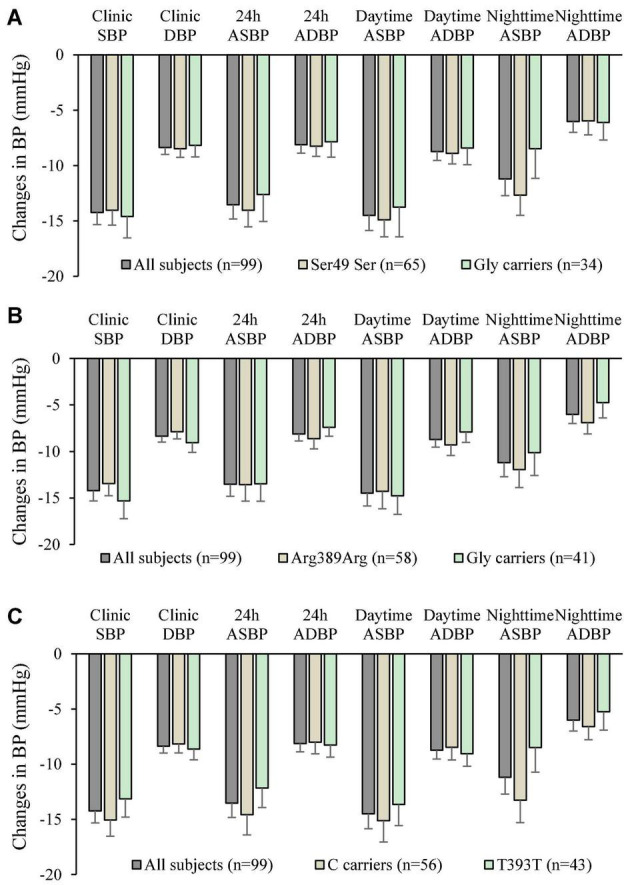
Changes in clinic and ambulatory blood pressure after 6 weeks treatment with bisoprolol 2.5 mg daily according to **(A)** *ADRB1* Ser49Gly genotypes, **(B)** *ADRB1* Arg389Gly genotypes, and **(C)** The G protein α-Subunit 1 gene (*GNAS*) 393C > T genotypes. Data are presented as mean ± SEM. There is no significant difference between groups (independent samples test was used).

**FIGURE 3:**
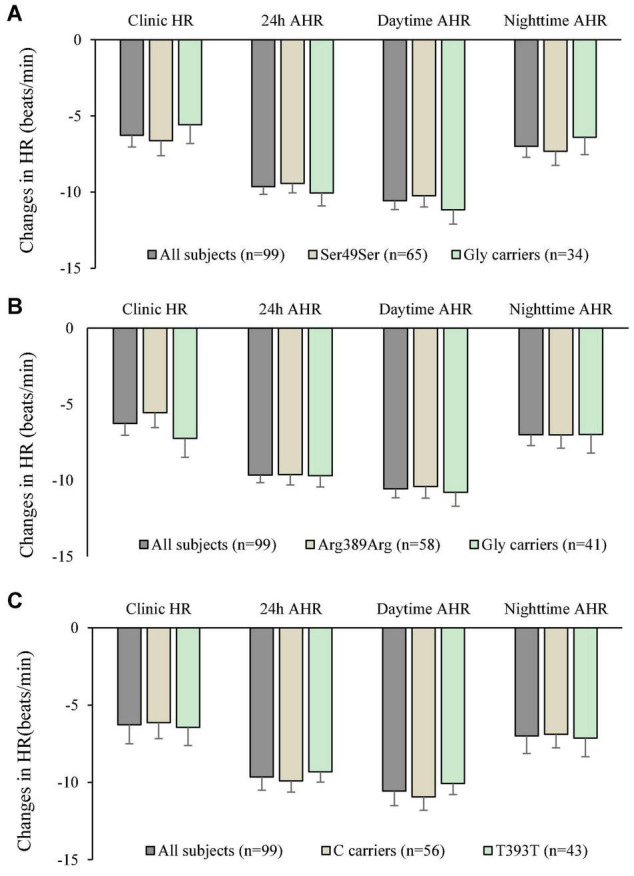
Changes in clinic heart rate (HR) and ambulatory heart rate (AHR) after 6 weeks treatment with bisoprolol 2.5 mg daily according to **(A)** *ADRB1* Ser49Gly genotypes, **(B)** *ADRB1* Arg389Gly genotypes, and **(C)** *GNAS* 393C > T genotypes. Data are presented as mean ± SEM. There is no significant difference between groups (independent samples test was used).

The genotype and allele frequencies of the 3 ploymorphisms studied are shown in Table 5. There were insufficient subjects with the homozygous genotype of the minor alleles to perform statistical tests on those groups separately and they were combined with the heterozygotes as carriers of the variant allele. The small number of subjects with the homozygous variants did not have extreme baseline BP and HR values or changes in BP and HR with bisoprolol treatment. There was no significant relationship between the genotypes of the 3 polymorphisms and baseline BP or HR. After 6 weeks of treatment with bisoprolol 2.5 mg daily, there was no significant difference in the clinic and ambulatory BP reductions among ADRB1 Ser49Gly, Arg389Gly and GNS1 393T > C genotypes comparing the wild type with variant carriers (Figures 2A–C). Similarly, no significant difference was observed in the clinic and ambulatory HR according to ADRB1 Ser49Gly, Arg389Gly and GNS1 393T > C genotypes (Figures 3A–C). Moreover, there was no significant difference in the clinic and ambulatory BP or HR reductions between the 4 ADRB1 diplotype groups (Gly49Arg389/Ser49Gly389, Ser49Arg389/Ser49Arg389, Ser49Arg389/Ser49Gly389 and Ser49Arg389/Gly49Arg389) (data not shown).

**TABLE 5:** Genotype and allele frequency of the studied SNPs (*n* = 99).

SNPs	Genotype/N (frequency)			Minor allele frequency
	Wt	Het	Mut	
*ADRB1* Ser49Gly	65 (0.657)	32 (0.323)	2 (0.020)	0.182
*ADRB1* Arg389Gly	58 (0.586)	37 (0.374)	4 (0.040)	0.227
*GNAS* 393C > T	6 (0.061)	50 (0.505)	43 (0.434)	0.313

*Wt, Het and Mut stand for wild type, heterozygous and mutant genotype, respectively. Minir allele frequency = total number of minor alleles/total number of alleles. All frequencies are in Hardy-Weinberg equilibrium by means of Chi-square test (P > 0.05).*

## Discussion

Primary hypertension is the most common cardiovascular disorder and a major risk factor for cardiovascular disease mortality (30, 31). It is a polygenic disease in which the genetic components interact with physiological and environmental factors. The RAAS represents a hormonal cascade in which PRA cleaves angiotensinogen into inactive Ang I, which undergoes further cleavage by the membrane-bound metalloproteinase angiotensin-converting enzyme to produce Ang II that is active in stimulating angiotensin receptors and aldosterone synthesis. The RAAS plays an essential role in the pathogenesis of hypertension (32). The present study found that the PAC was related to baseline DBP and plasma potassium. The HR-lowering effects of bisoprolol treatment correlated significantly with baseline PRA but not with baseline PAC and Ang II levels.

It was reported that patients with higher baseline PRA would have a greater reduction in BP with RAAS-blocking drugs than patients with lower PRA (33). β-Blockers can reverse the neurohumoral effects of the sympathetic nervous system and lower PRA with potential ensuing symptomatic and prognostic benefits, and thus remain an option for the first-line antihypertensive treatment in several guidelines. In this study, bisoprolol, a β1-Selective non-vasodilatory β-Blocker, decreased PRA, PAC, and Ang II levels, which is consistent with previous studies with nebivolol, another β1-Selective but vasodilatory β-Blocker, and with other β-Blockers (14, 34, 35).

Consistent with some previous reports, we did not find a better BP response to the β-Blocker in individuals homozygous for either the ADRB1 Arg389Gly or Ser49Gly polymorphisms (36, 37). However, a recent study reported that patients with the ADRB1 homozygous form showed a better response to metoprolol compared with those harboring the heterozygous variant (38). Suonsyrja et al. (36) reported that patients with the Gly389Gly genotype tended to better BP response to bisoprolol than those with Arg389Arg genotypes and no significant difference was found. Johnson et al. (22) reported a more than 2-fold greater percentage reduction in 24 h DBP in Arg389 homozygotes than in Gly389 carriers and a nearly 3-fold greater percentage reduction in daytime DBP after metoprolol treatment in 40 hypertensive subjects, including 10 African Americans. In addition to codon 389, we evaluated the impact of codon 49 genotype and the codon 49/389 haplotype on the antihypertensive response to bisoprolol. Although the functional data for the codon 49 variant indicate that the effect of this polymorphism is primarily on receptor regulation, with the Gly49 allele undergoing greater agonist-mediated receptor down regulation *in vitro*, we did not find a greater antihypertensive response to bisoprolol in the Ser49 homozygotes. Johnson et al. (22) also investigated the ADRB1 haplotypes and found the subjects with the Ser49Arg389/Ser49Arg389 diplotype showed a decrease in DBP of 14.7 mm Hg versus 0.5 mm Hg in patients with the Gly49Arg389/Ser49Gly389 diplotype. However, in our study, there was no significant difference in the reduction of BP and HR between the Gly49Arg389/Ser49Gly389 diplotype, Ser49Arg389/Gly49Arg389 and the Ser49Arg389/Ser49Gly389 diplotype subjects after bisoprolol treatment. Moreover, there was a study that showed Arg389-homozygous subjects had a greater response in resting SBP after a single dose of atenolol compared to Gly389 homozygotes (39).

In white subjects, a common silent polymorphism (393C > T) involving a change in codon 131 from ATT (Ile) to ATC (Ile) in the GNAS gene was linked to hypertension and the BP response to β-Blocker therapy (24). Another study in 2,308 Japanese individuals found a marginally significant difference in the frequencies of the alleles and genotypes between the hypertensives and normotensives, which was more significant in non-heavy smokers (40). In our study, there were 8 (8%) patients who were smokers, and all of them were non-heavy smokers. However, we did not find any relationship between the GNAS 393C > T polymorphism and BP or HR changes with bisoprolol treatment in our study. Differences between the present study and previous reports may be related to the subjects’ ethnicity or the type and duration of β-Blocker therapy, or various other factors.

This study had several limitations. Firstly, the number of subjects in this study is not large enough to detect minor effects of the RAAS parameters or genotypes. Secondly, we only examined some common polymorphisms in ADRB1 and GNAS and the number of subjects homozygous for the minor alleles of each polymorphism was insufficient to assess the responses in those groups separately. Lastly, we cannot exclude a small effect of these genotypes due to the relatively small sample size. We have previously reported that plasma concentrations of bisoprolol and polymorphisms in the drug metabolizing enzymes CYP2D6 and CYP3A5 did not influence the BP and HR response to bisoprolol in these subjects.

## Conclusion

Baseline values of PRA, PAC, and Ang II were not associated with the BP response to bisoprolol treatment, but bisoprolol treatment had a suppressive effect on all these parameters. The common Ser49Gly and Arg389Gly polymorphisms in the ADRB1 gene had no significant association with the reduction of the clinic and ambulatory BP and HR after bisoprolol treatment. There was no significant effect of the GNAS T393C polymorphisms on BP and HR changes with bisoprolol treatment.

## Data Availability Statement

The original contributions presented in the study are included in the article/supplementary material, further inquiries can be directed to the corresponding authors.

## Ethics Statement

The studies involving human participants were reviewed and approved by the Joint Clinical Research Ethics Committee of The Chinese University of Hong Kong and New Territories East Cluster (CUHK-NTEC) with reference number CRE-2011.616-T. The patients/participants provided their written informed consent to participate in this study.

## Author Contributions

WZ and SC analyzed the data and wrote this manuscript. BT and TC designed the research project. TC and WZ included the patients and followed this study. CL, AC, and CH performed the experiments. BT, AK, and CH revised this manuscript. All authors contributed to the article and approved the submitted version.

## Conflict of Interest

The authors declare that the research was conducted in the absence of any commercial or financial relationships that could be construed as a potential conflict of interest.

## Publisher’s Note

All claims expressed in this article are solely those of the authors and do not necessarily represent those of their affiliated organizations, or those of the publisher, the editors and the reviewers. Any product that may be evaluated in this article, or claim that may be made by its manufacturer, is not guaranteed or endorsed by the publisher.
